# The Carcinogenicity of Alendronate in Patients with Osteoporosis: Evidence from Cohort Studies

**DOI:** 10.1371/journal.pone.0123080

**Published:** 2015-04-16

**Authors:** Ling-Xiao Chen, Guang-Zhi Ning, Zhi-Rui Zhou, Yu-Lin Li, Di Zhang, Qiu-Li Wu, Tian-Song Zhang, Lei Cheng, Shi-Qing Feng

**Affiliations:** 1 Department of Orthopaedics, Tianjin Medical University General Hospital, 154 Anshan Road, Heping District, Tianjin, 300052, People’s Republic of China; 2 Department of Radiation Oncology, Fudan University Shanghai Cancer Center, Shanghai, 200032, People’s Republic of China; 3 Department of Oncology, Shanghai Medical College, Fudan University, Shanghai, 200032, People’s Republic of China; 4 Internal Medicine of Traditional Chinese Medicine Department, Jing 'an District Central Hospital of Shanghai, NO. 259, Xikang Road, 200040, Shanghai, People’s Republic of China; 5 Comprehensive Cancer Center of Drum-Tower Hospital, Medical School of Nanjing University, Clinical Cancer Institute of Nanjing University, 321 Zhongshan Road, Nanjing, 210008, People’s Republic of China; Baylor University Medical Center, UNITED STATES

## Abstract

**Context:**

Alendronate may relate to the incidence of cancers, especially esophageal and colon cancer. But the results are inconsistent in different studies.

**Objective:**

To quantify the association between the use of alendronate and the occurrence of different types of cancer.

**Data Sources:**

We searched Embase, Pubmed, CENTRAL, SIGLE and clinicaltrials.gov, up to 2014 June.

**Study Selection:**

Cohort studies reporting association between alendronate or bisphosphonate therapy including alendronate in patients with osteoporosis and risk of cancer were selected by two authors.

**Data Extraction:**

Two authors independently extracted the data. The Chi-square test and the I-square test were used for testing heterogeneity between studies.

**Data Synthesis:**

Eight cohort studies were included in the meta-analysis. Meta-analysis result manifested that alendronate significantly increased the incidence of lung cancer (HR 1.23, 95%CI 1.03 to 1.47, P value = 0.03), nevertheless, there was no significant difference after we excluded either Lee’s 2012 study (HR 1.17, 95%CI 0.95 to 1.44, P value = 0.13) or Chiang’s 2012 study (HR 1.47, 95%CI 1 to 2.17, P value = 0.05). For the incidence of colorectal cancer, no significant difference occurred (HR 0.91, 95%CI 0.74 to 1.13, P value = 0.39), but there was a positive relationship when we used fixed model (HR 0.85, 95%CI 0.78 to 0.93, P value = 0.004). For the incidence of liver cancer, there was no significant difference (HR 1.36, 95%CI 0.9 to 2.04, P value = 0.14), however, the result changed after we excluded Chiang’s 2012 study (HR 1.69, 95%CI 1.03 to 2.77, P value = 0.04). There was no significant difference in other types of cancer.

**Conclusion:**

Based on current evidences, alendronate therapy may be associated with a high risk of lung cancer, may with an excess risk of liver cancer, a low risk of colorectal and no related risk of other cancers.

## Introduction

Because of its effectiveness and low cost, alendronate is recommended as the first-line drug in the treatment of osteoporosis in postmenopausal women, older men and patients with glucocorticoid-induced osteoporosis [1–3]. In addition, it is used in the secondary prevention of osteoporotic fractures in postmenopausal women [4]. Alendronate has the ability to inhibit the activity of osteoclasts and decrease the bone turnover rate. However, its use is associated with the potential risks of upper gastrointestinal bleeding or ulcers and rare cases of cancer.

In recent years, several researchers have reported that the use of bisphosphonates (including alendronate) is related to the incidence of cancers, especially esophageal and colon cancers. However, the results are inconsistent. For example, some studies have shown that bisphosphonate use is associated with a high risk of the occurrence of esophageal cancer [5, 6], but no significant differences in the increasing risk was observed in other research [7, 8]. Another paradox is that although one study indicated that alendronate significantly reduced the incidence of colon cancer [9]; another study reported that low doses of alendronate significantly increased the incidence of colon cancer, while the results were reversed at high doses [5]; however, other studies have shown that there was no significant difference.

The relationship between the usage of alendronate and the incidence of cancers is important for guiding patients in the selection of osteoporosis treatments. Therefore, we performed this meta-analysis and systematic review of cohort studies to quantify the association between the use of alendronate and the occurrence of different types of cancer.

## Methods

### Criteria for considering studies

The studies were considered to be acceptable for inclusion in this article if they met the following criteria: (1) Participants: patients with osteoporosis; (2) Interventions and comparisons: alendronate or bisphosphonate therapy, including alendronate vs control groups; (3) Outcomes: the incidence of cancer (all-cause cancer, colorectal cancer, gastric cancer, esophageal cancer, liver cancer, pancreatic cancer, lung cancer, breast cancer, cervical cancer, bladder cancer, kidney cancer, oral cancer, ovarian cancer, endometrial cancer, prostate cancer, lymphoma, bile duct cancer and small intestine cancer); and (4) Study design: cohort studies.

Trials were excluded if they (1) were abstracts, letters, or proceedings of meetings; (2) had repeated data or did not report outcomes of interest; (3) did not supply sufficient data about alendronate; or (4) were case-control studies.

### Search strategy and study selection

A Meta-analysis of Observational Studies in Epidemiology (MOOSE) [10] was used to perform this systematic review. We searched Embase (from 1974 to June 2014), PubMed (From 1966 to June 2014), the Cochrane Central Register of Controlled Trials (CENTRAL), SIGLE (System for Information on Grey Literature in Europe) and clinicaltrials.gov. Keywords and MeSH terms including “colorectal cancer”, “gastric cancer”, “esophagus cancer”, “liver cancer”, “pancreas cancer”, “lung cancer”, “breast cancer”, “cervical cancer”, “bladder cancer”, “kidney cancer”, “oral cancer”, “ovary cancer”, “endometrial cancer”, “prostate cancer”, “lymphoma”, “bile duct cancer”, “small intestine cancer”, “bisphosphonate”, “alendronate”, “osteoporosis”, “cohort” and “follow-up studies” were used in the search strategy. We also reviewed the reference list of each of the included studies for any relevant papers that had been omitted from the search.

Two authors independently performed the selections based on the title and abstract. Any disagreement between the two authors was resolved by a discussion. If there was no consensus, a third reviewer (Feng) was consulted. If two or more studies used the data from the same cohort, we included the newest and most detailed study.

### Data extraction and quality assessment

Information about the study, country, sex, sample size, age, duration of follow-up, covariates in the adjusted model, measure of associations, categories of dosage and types of cancer were extracted for each included study. One of the authors entered the data into RevMan 5.3, after which another author checked all of the values. We used the Newcastle-Ottawa Scale (NOS) for assessing the quality of the studies included by the two authors. The scale, which has a total score of up to nine, includes three categories (selection, comparability and outcome) and eight entries (representativeness of the exposed cohort, selection of the nonexposed cohort, ascertainment of exposure, demonstration that outcome of interest was not present at start of study, comparability of cohorts on the basis of the design or analysis, assessment of outcome, was follow-up long enough for outcomes to occur and adequacy of follow-up of cohorts).

### Statistical methods

We made meta-analyses using Stata software (version 12.0, StataCorp, College Station, TX). Hazard ratios (HR) and 95% confidence intervals (CI) were used as a measure of the incidence of the cancers. The term DDD was used to indicate the defined daily dose, which has been proposed by WHO as a statistical measure of drug consumption. For alendronate, the value of the DDD was 10 mg. The Chi-square test and the I-square test were used to test the heterogeneity between the studies. If heterogeneity was not present (P>0.10, I^2^<50%), a fixed-effect model was adopted for the analysis; otherwise, the random-effect model was employed. Additionally, I^2^ was used to evaluate the heterogeneity (an I^2^ of more than 50% was considered to be high heterogeneity and an I^2^ of less than 25% was considered to be no heterogeneity[11]). If the I^2^ was more than 50%, sensitivity analyses were used to investigate the origin of the heterogeneity. The funnel plot was used to identify a possible publication bias if the number of studies was larger than 10.

There was no protocol.

## Results

### Study Identification and selection

**Fig 1:**
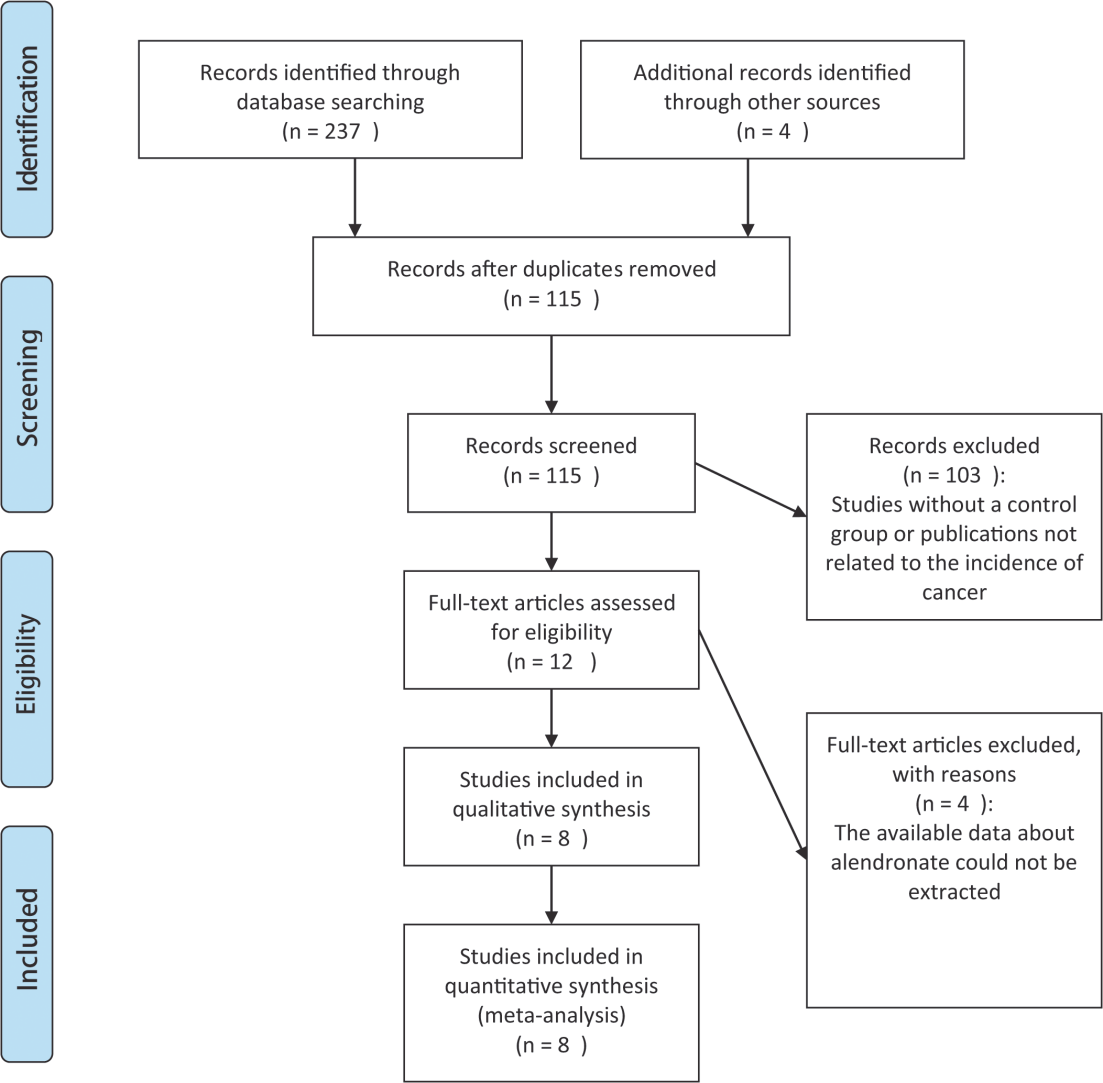
PRISMA flow diagram for study identification and inclusion.

The PRISMA flow diagram of the selection of the studies is depicted in Fig 1. The search was performed on June 8th, 2014, and 237 records were identified in the primary search and 4 records were identified from other sources. After the removal of 126 duplicate references, 115 records were screened. Twelve of the publications were eligible for inclusion, and the others were not selected for various reasons (e.g., studies without a control group or publications not related to the incidence of cancer). A total of 8 studies were included in the qualitative synthesis, and the data on these studies were included in the meta-analysis [5, 9, 12–17]. Four of the studies were excluded because the available data on alendronate could not be extracted [6–8, 18].

### Study characteristics

Table 1 provides a summary of the studies included in the review. The duration of the follow-up ranged from 2.8 years to 12 years. Five of the studies included women, and the other studies included both men and women. Four studies were conducted in Denmark, two in Taiwan, one in the UK and one in the USA. The dates of publication were between 2010 and 2013. Three of the studies that included men and women had been adjusted for gender. Five of the studies were adjusted for age, and in two studies, no adjustment was made because all of the participants were the same age. Four of the studies were adjusted for NSAID prescriptions, four for alcohol consumption, two for proton pump inhibitor (PPI) use, two for the amount of prednisolone administered, two for the Charlson index and two for BMI. Other adjust factors included known ulcerative colitis, known Crohn’s disease, known coeliac disease, smoking, Barretts esophagus, working or not, married or not, income above vs. below median (112,000 DKK/year), gastric surgery before, education, irradiation, chemotherapy, physical activity, estrogen-only use, estrogen-progestin use, history of endoscopy, history of mammography, total calcium intake, total vitamin D intake, and 5-year hip fracture probability and they were referred only one time.

**Table 1:** Characteristics of studies included in meta-analysis.

Study	Country	Sex	Sample size	Age Years^b^	Follow-up Years^b^	NOS	Types of cancer
Abrahamsen 2012^a^	Denmark	W	153030	71.9 (10)	3.5 (1–11)	6	1–3
Cardwell 2010	UK	M/W	81369(py)	70 (11.4)	4.5(2.6)/4.4(2.6)	8	1–2
Chiang 2012	Taiwan	W	27603	73.4 (8.4)/73.5 (8.4)	4.3(2.5)/4.9(2.6)	6	1–8
Lee 2012	Taiwan	M/W	21918	Not mentioned	2.92/3.04	7	1–7
Vestergaard (A) 2011	Denmark	M/W	215142	70.5 (11.4)	2.8	6	1、3–4、8
Passarelli 2013	USA	W	102219(py)	63.1 (7.2)/67.2 (6.4)	12	8	3
Vestergaard(B) 2011	Denmark	W	306494	71.7 (10.7)	Not mentioned	6	6

W: women, M: man, py: person years, NOS: Newcastle-Ottawa Scale, 1: Esophageal, 2: gastric, 3: colorectal, 4: liver, 5: lung, 6: breast, 7: cervical, 8: pancreatic.

For age and follow up, the left of the oblique line represents alendronate group and the right represents control group.

^a^Pazianas 2012 is the other publication of the study.

^b^ For years, the numbers in the parenthesis represent standard deviation or range.

The average score of the studies’ quality was 6.7, and all of the studies had a score greater than 6. Two studies were regarded as having mild selection bias [15, 16]. Two of the studies were considered as having moderate comparability because they were only adjusted for two factors [13, 14]. Four included studies were judged as having mild outcome bias [5, 14–16].

### Incidence of lung cancer

**Fig 2:**
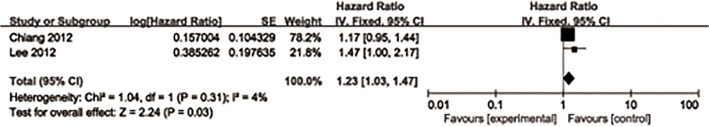
Forest plot of the incidence of lung cancer.

The incidence of lung cancer was investigated in two studies, with a total of 49521 participants and 609 cancer patients. The pooled estimate of the two studies [13, 14] indicated that the incidence of lung cancer significantly increased in the alendronate groups in comparison with the control groups (HR 1.23, 95%CI 1.03 to 1.47, P value = 0.03, I^2^ = 4%) (Fig 2).

Lee et al. [13] reported that there was a strong positive relationship between the use of alendronate and the incidence of lung cancer (HR 3.07, 95%CI 1.97 to 4.76, P value<0.001) when the dosage was ≥1.0 g/per year. There was no significant difference in the other dosage amounts.

### Incidence of all-cause cancer

The pooled estimate of four studies [13–15, 17] indicated that there was no significant difference in the incidence of all-cause cancer between the alendronate groups and the control groups (HR 0.94, 95%CI 0.78 to 1.12, P value = 0.48, I^2^ = 73%) (S1 Fig).

Lee et al. [13] indicated that there was a strong positive relationship between the use of alendronate and the incidence of all-cause cancer (HR 1.69, 95%CI 1.39 to 2.04, P value<0.001) when the dosage was ≥1.0 g/per year. However, Abrahamsen et al. [15] showed that the use of alendronate significantly decreased the incidence of all-cause cancer (HR 0.44, 95%CI 0.27 to 0.69) when the number of prescriptions were ≥10. There was no significant difference for the other types of dosages.

### Incidence of colorectal cancer

The pooled estimate of five studies [5, 9, 12–14] indicated that there was no significant difference in the incidence of colorectal cancer (HR 0.91, 95%CI 0.74 to 1.13, P value = 0.39, I^2^ = 80%) (S2 Fig).

Vestergaard et al. [5] showed that the use of alendronate significantly increased the incidence of colorectal cancer at a low daily dosage (DDD≤0.66, HR 1.44, 95%CI 1.08 to 1.93) and at a medium daily dosage (DDD = 0.67–0.99, HR 1.49, 95%CI 1.08 to 2.04); however, alendronate significantly decreased the incidence of colorectal cancer at high dosages (DDD≥1, HR 0.3, 95%CI 0.14 to 0.63). Vestergaard et al. [5] also reported that the use of alendronate significantly increased the incidence of colorectal cancer when the accumulated dose was ≤365 DDD (HR 1.49, 95%CI 1.07 to 2.06). There was no significant difference in the other types of dosages.

### Incidence of gastric cancer

The pooled estimate of four studies [5, 13–15] indicated that there was no significant difference in the incidence of gastric cancer (HR 0.86, 95%CI 0.67 to 1.10, P value = 0.22, I^2^ = 24%) (S3 Fig).

Vestergaard et al. [5] reported that the use of alendronate significantly increased the incidence of gastric cancer when the accumulated dose ranged from 366 DDD to 730 DDD (HR 3.11, 95%CI 1.03 to 9.34). There was no significant difference in the other types of dosages.

### Incidence of esophagus cancer

The pooled estimate of five studies [5, 13–15, 17] indicated that there was no significant difference in the incidence of esophageal cancer (HR 1.07, 95%CI 0.7 to 1.64, P value = 0.75, I^2^ = 52%) (S4 Fig).

Vestergaard et al. [5] showed that the use of alendronate significantly increased the incidence of esophageal cancer at a low dosage (DDD≤0.66, HR 3.19, 95%CI 1.4 to 7.29). Moreover, Vestergaard et al. [5] reported that the use of alendronate significantly increased the incidence of esophageal cancer when the accumulated dose was ≤365 DDD (HR 4.45, 95%CI 1.93 to 10.3). There was no significant difference in the other types of dosages.

### Incidence of liver cancer

The incidence of liver cancer was investigated in three studies [5, 13, 14], with a total of 264663 participants and 529 cancer patients. The pooled estimate of the three studies indicated that there was no significant difference in the incidence of liver cancer (HR 1.36, 95%CI 0.9 to 2.04, P value = 0.14, I^2^ = 65%) (S5 Fig).

Lee et al. [13] indicated that there was a positive relationship between the use of alendronate and the incidence of liver cancer (HR 1.94, 95%CI 1.16 to 3.24, P value<0.05) when the dosage was ≥1.0 g/per year. In addition, Vestergaard et al. [5] showed that the use of alendronate significantly increased the incidence of liver cancer at a low dosage (DDD≤0.66, HR 4.13, 95%CI 1.66 to 10.3). Vestergaard et al. [5] reported that the use of alendronate significantly increased the incidence of liver cancer when the accumulated dose was ≤365 DDD (HR 5.53, 95%CI 2.13 to 14.3). There was no significant difference in the other types of dosages.

### Incidence of pancreas cancer

The pooled estimate of two studies [5, 14] indicated that there was no significant difference in the incidence of pancreatic cancer between the alendronate groups and the control groups (HR 1.11, 95%CI 0.78 to 1.59, P value = 0.56, I^2^ = 7%) (S6 Fig).

Vestergaard et al. [5] reported that the use of alendronate significantly increased the incidence of pancreatic cancer when the accumulated dose was ≤365 DDD (HR 5.53, 95%CI 2.13 to 14.3) and ranged from 366 DDD to 730 DDD (HR 2.82, 95%CI 1.25 to 6.35). There was no significant difference in the other types of dosages.

### Incidence of breast cancer

The pooled estimate of three studies [5, 13, 14] indicated that there was no significant difference in the incidence of breast cancer (HR 0.92, 95%CI 0.82 to 1.02, P value = 0.12, I^2^ = 0%) (S7 Fig).

Vestergaard et al. [16] indicated that alendronate significantly reduced the incidence of breast cancer when the treatment time ranged from 1.1 years to 5 years (HR 0.84, 95%CI 0.72 to 0.98, P value<0.05), with no significant effect when the treatment time was more than five years or less than one year. There was no significant difference in the other types of dosages.

### Incidence of cervical cancer

The pooled estimate of two studies [13, 14] indicated that there was no significant difference in the incidence of cervical cancer (HR 0.79, 95%CI 0.52 to 1.18, P value = 0.24, I^2^ = 27%) (S8 Fig). There was no significant difference in any of the types of dosages.

### Incidence of bladder and kidney cancer

The pooled estimate of two studies [13, 14] indicated that there was no significant difference in the incidence of bladder and kidney cancer (HR 0.98, 95%CI 0.69 to 1.40, P value = 0.92, I^2^ = 46%) (S9 Fig). There was no significant difference in any of the types of dosages.

### Other cancers

#### Lee 2012

There was no significant difference between the alendronate group and the control group in the incidence of oral cancer (HR 0.4, 95%CI 0.09 to 1.74), ovarian cancer (HR 0.58, 95%CI 0.07 to 4.93), endometrial cancer (HR 1.79, 95%CI 0.43 to 7.49), prostate cancer (HR 1.83, 95%CI 0.92 to 4.65), lymphoma (HR 2.34, 95%CI 0.93 to 5.94) or other cancers (HR 1.03, 95%CI 0.73 to 1.45).

There was a positive relationship between the two groups in the incidence of prostate cancer (HR 3.25, 95%CI 1.43 to 7.36, P value<0.01) and lymphoma (HR 4.37, 95%CI 1.49 to 12.8, P value<0.01) when the dosage was ≥1.0 g/per year. There was no significant difference in the other types of dosages.

#### Vestergaard 2011

There was no significant difference between the alendronate group and the control group in the incidence of bile duct cancer (HR 1.88, 95%CI 0.76 to 4.68) or small intestine cancer (HR 2.19, 95%CI 0.46 to 10.4).

The use of alendronate significantly increased the incidence of bile duct cancer (HR 3.11, 95%CI 1.03 to 9.34) and cancer of the small intestine (HR 10.5, 95%CI 1.75 to 63.2) when the accumulated dose ranged from 366 DDD to 730 DDD. There was no significant difference in the other types of dosages.

### Sensitivity analyses and publication bias

Overall, most of the outcomes were stable. For the incidence of colorectal cancer, there was a positive relationship when we used a fixed model (HR 0.85, 95%CI 0.78 to 0.93, P value = 0.004). For the incidence of liver cancer, the result changed after we excluded Chiang’s 2012 study (HR 1.69, 95%CI 1.03 to 2.77, P value = 0.04). The details of the sensitivity analyses are shown in S1 Table. We were unable to perform a funnel plot because the number of included studies was less than 10.

## Discussion

### Summary of main results

This article shows the positive relationship between the use of alendronate and lung cancer. The sensitivity analyses indicate that there may be a positive relationship between the use of alendronate and colorectal or liver cancer. There was no significant difference between the alendronate group and the control group in the incidence of other cancers.

### Agreements and disagreements in the current literature

For esophageal cancer, our study results are consistent with the previous meta-analyses. Two previous meta-analyses, including cohort and case-control studies, showed that alendronate was not significantly associated with the incidence of esophageal cancer [19, 20]. Moreover, one case-control study that was conducted in Taiwan [21] reported a negative result (Odds Risk 0.61, 95%CI 0.21 to 1.75), and one nested case-control study conducted in the UK [22] indicated the same result in the data from the QResearch primary care database (Adjusted Odds Risk 0.91, 95%CI 0.73 to 1.14, P value = 0.4), the data from Clinical the Practice Research Datalink (CPRD) (Adjusted Odds Risk 1.03, 95%CI 0.83 to 1.28, P value = 0.8) and in the combined data (Adjusted Odds Risk 0.97, 95%CI 0.83 to 1.13, P value = 0.7).

For gastric cancer, there was no significant association between alendronate and gastric cancer in the two case-control studies, the data of which were obtained from the UK’s primary care electronic health records (CPRD)[22, 23]. Our results are agreed with the results from the CPRD. However, the result from the QResearch primary care database showed a positive relationship (Adjusted Odds Risk 1.47, 95%CI 1.11 to 1.95, P value = 0.008). Because the baseline characteristics of the CPRD and QResearch were almost the same, the difference between the CPRD and QResearch may be coincidental.

For colorectal cancer, one UK study [22] reported data from the QResearch primary care database (Adjusted Odds Risk 1.1, 95%CI 0.98 to 1.22, P value = 0.1), data from the Clinical Practice Research Datalink (CPRD) (Adjusted Odds Risk 1.1, 95%CI 0.98 to 1.22, P value = 0.1) and combined data (Adjusted Odds Risk 1.1, 95%CI 1.01 to 1.19, P value = 0.02). However, it was not significant at the 1% level. Our results basically support these results. Conversely, one meta-analysis that contained three case-control studies and one cohort study showed that oral bisphosphonates were associated with a reduced risk of colorectal cancer (Adjusted Odds Risk 0.87, 95%CI 0.78 to 0.97) [24]. Another meta-analysis containing six population-based observational studies also indicated the reduced risk for colorectal cancer (Adjusted Odds Risk 0.85, 95%CI 0.74 to 0.98) [25]. However, the two meta-analyses included two or more types of bisphosphonates that were not able to be separated. As a result, we could not exclude the possibility that other bisphosphonates and not the alendronate contributed to the positive association.

For breast cancer, there is an inconsistency among our study results and other researches. A meta-analysis that contained two cohort studies and two case-control studies indicated that the use of bisphosphonates, including alendronate, significantly decreased the incidence of breast cancer when compared with nonusage (Risk Ratio 1.24, 95%CI 0.74 to 0.98) [26]. However, a randomized controlled study showed that there was no significant difference between the alendronate group and the placebo group (HR 1.24, 95%CI 0.84 to 1.83) [27]. Our results agreed with the results from the randomized controlled study. The positive result in a previous meta-analysis might be attributed to the two following causes: 1. one of the cohort studies contained many types of bisphosphonates, of which etidronate and not alendronate had a positive effect; 2. the result of the case-control studies may have benefited the treatment group.

For lung cancer, a vitro study by Lin et al. [28] found that alendronate could selectively inhibit the proliferation of lung cancer cells. No other related studies were searched. However, our results showed that there was a positive relationship between the use of alendronate and the incidence of lung cancer. The mechanism is unclear now and relevant researchers should pay attention to it.

For the other types of cancer, this is the first attempt to integrate the available data and draw conclusions.

### Strengths and weaknesses

The strengths of this paper are (1) we used a comprehensive search strategy to minimize the possibilities of publication bias; (2) sensitivity analyses were conducted to investigate the origin of the high heterogeneity; and (3) the total sample size was large and the duration of the follow-up was sufficient.

However, the results of this article should be interpreted with some limitations. First, the observational studies had some unmeasured confounding factors. Second, two of the included studies were adjusted only for age and sex, which may have overestimated the incidence of cancer because there was a positive relationship between some of the other factors (i.e., smoking and alcohol) and the incidence of cancer. Third, the geographical location of the included studies (only four countries, all of which were located in North America, Europe and East Asia) was limited, which reduces the available scope of our research. Fourth, there were differences in the categories of the dosages, resulting in our inability to integrate them and determine whether there was a dose-response reaction or not. Fifth, for lung cancer, two related studies were conducted in Taiwan, and the duration of the study time was almost the same. As we could not exclude that a possible overlap existed in the included patients, we conducted a sensitivity analysis to test the robustness of the result (S1 Table.). The result changed and showed no significant difference after excluding either Lee’s 2012 study (HR 1.17, 95%CI 0.95 to 1.44, P value = 0.13) or Chiang’s 2012 study (HR 1.47, 95%CI 1 to 2.17, P value = 0.05). From this information, we concluded that it was not a stable result and should be carefully interpreted.

## Conclusion

This meta-analysis provides evidence that the use of alendronate may be associated with a high risk of occurrence of lung cancer, may increase the incidence of liver cancer and may decrease the incidence of colorectal cancer. There may be no relationship between the use of alendronate and the incidence of other cancers. Future studies should focus on the dose-response and the duration of the treatment time with alendronate and the incidence of the cancers, especially lung cancer, liver cancer and colorectal cancer. Additional high-quality RCTs are needed to estimate the carcinogenicity of alendronate.

## Supporting Information

S1 Fig Forest plot of the incidence of all-cause cancer. (EPS)

S2 Fig Forest plot of the incidence of colorectal cancer. (EPS)

S3 Fig Forest plot of the incidence of gastric cancer. (EPS)

S4 Fig Forest plot of the incidence of esophagus cancer. (EPS)

S5 Fig Forest plot of the incidence of liver cancer. (EPS)

S6 Fig Forest plot of the incidence of pancreas cancer. (EPS)

S7 Fig Forest plot of the incidence of breast cancer. (EPS)

S8 Fig Forest plot of the incidence of cervical cancer. (EPS)

S9 Fig Forest plot of the incidence of bladder and kidney cancer. (EPS)

S1 File PRISMA Checklist for this meta-analysis. (DOC)

S1 Table Sensitivity analyses. (DOCX)
