# Supplementary material for: The Carcinogenicity of Alendronate in Patients with Osteoporosis: Evidence from Cohort Studies
Source: PLoS One. 2015 Apr 16;10(4):e0123080. doi: 10.1371/journal.pone.0123080 (PMC4399980; doi:10.1371/journal.pone.0123080)
Supplement: S1 Table — (DOCX) [file pone.0123080.s011.docx]

S1 Table. Sensitivity analyses.

| Sensitivity analysis | Heterogeneity | | Effect | |
| --- | --- | --- | --- | --- |
|  | I^2^ | Chi^2^ | HR and 95%CI | P |
| All-cause cancer  All studies (random model)  All studies (fixed model)  Exclude Abrahamsen 2012  Exclude Cardwell 2010  Exclude Chiang 2012  Exclude Lee 2012  Colorectal cancer  All studies (random model)  **All studies (fixed model)**  Exclude Chiang 2012  Exclude Lee 2012  Exclude Passarelli 2013  Exclude Pazianas 2012  Exclude Vestergaard (A) 2011  Esophagus cancer  All studies (random model)  All studies (fixed model)  Exclude Abrahamsen 2012  Exclude Cardwell 2010 | 73%  73%  5%  79%  80%  80%  80%  **80%**  82%  84%  85%  31%  73%  52%  52%  50%  55% | 10.94  10.94  2.10  9.32  9.98  10.14  19.83  **19.83**  16.32  19.28  19.81  4.34  10.96  8.39  8.39  6.04  6.69 | 0.94 (0.78, 1.22)  1.03 (0.96, 1.10)  1.05 (0.97, 1.13)  0.96 (0.79, 1.17)  0.84 (0.58, 1.12)  0.83 (0.58, 1.18)  0.91 (0.74, 1.13)  **0.85 (0.78, 0.93)**  0.89 (0.69, 1.14)  0.90 (0.71, 1.15)  0.93 (0.71, 1.23)  0.99 (0.86, 1.14)  0.85 (0.70, 1.04)  1.07 (0.70, 1.64)  0.99 (0.75, 1.31)  1.24 (0.75, 2.06)  1.22 (0.71, 2.12) | 0.48  0.43  0.20  0.69  0.34  0.30  0.39  **0.004**  0.35  0.4  0.63  0.91  0.12  0.75  0.95  0.41  0.47 |
| Exclude Chiang 2012  Exclude Lee 2012  Exclude Vestergaard (B) 2011  Liver cancer  All studies (random model)  All studies (fixed model)  **Exclude Chiang 2012**  Exclude Lee 2012  Exclude Vestergaard (A) 2011  Lung cancer  **All studies (fixed model)**  **Exclude Chiang 2012**  Exclude Lee 2012 | 54%  64%  18%  65%  65%  **31%**  76%  56%  **4%**  /  / | 6.51  8.36  3.66  5.66  5.66  **1.44**  4.16  2.25  **1.04**  /  / | 0.99 (0.60, 1.61)  1.08 (0.67, 1.75)  0.89 (0.63, 1.25)  1.36 (0.90, 2.04)  1.18 (0.97, 1.43)  **1.69 (1.03, 2.77)**  1.48 (0.62, 3.52)  1.18 (0.85, 1.64)  **1.23 (1.03, 1.47)**  **1.47 (1, 2.17)**  1.17 (0.95, 1.44) | 0.96  0.75  0.51  0.14  0.09  **0.04**  0.38  0.32  **0.03**  **0.05**  0.13 |
